# Clinical characteristics and evaluation of LDL-cholesterol treatment of the Spanish Familial Hypercholesterolemia Longitudinal Cohort Study (SAFEHEART)

**DOI:** 10.1186/1476-511X-10-94

**Published:** 2011-06-10

**Authors:** Nelva Mata, Rodrigo Alonso, Lina Badimón, Teresa Padró, Francisco Fuentes, Ovidio Muñiz, Francisco Perez-Jiménez, José López-Miranda, Jose L Díaz, Jose I Vidal, A Barba, Mar Piedecausa, Juan F Sanchez, Luis Irigoyen, Eliseo Guallar, José M Ordovas, Pedro Mata

**Affiliations:** 1 Department of Epidemiology, Madrid Health Authority and Fundación Hipercolesterolemia Familiar, Madrid, Spain; 2 Lipid, Clinic, Internal Medicine Department. IIS-Fundación Jiménez Díaz, Madrid; 3 Centro de Investigación Cardiovascular CSIC-ICCC, Hospital Sant Pau and IIB-Sant Pau, and CIBEROBN, ISC III, Barcelona, Spain; 4 Lipid Unit, IMIBIC/Hospital Reina Sofía and CIBEROBN, ISC III, Cordoba; 5 Internal Medicine Department. Hospital Virgen del Rocío, Sevilla, Spain; 6 Internal Medicine Department. Hospital Abente y Lago, A Coruña, Spain; 7 Endocrinology Department, Hospital de Lugo, Spain; 8 Internal Medicine Department. Hospital General de Albacete, Spain; 9 Internal Medicine Department. Hospital de Elche, Spain; 10 Internal Medicine Department. Hospital de Cáceres, Spain; 11 Endocrinology Department. Hospital de Vitoria, Spain; 12 Departments of Epidemiology and Medicine. Johns Hopkins Bloomberg School of Public Health, Baltimore, MD, USA; 13 Centro Nacional de Investigaciones Cardiovasculares (CNIC), Madrid, Spain; 14 Nutrition and Genomics Laboratory, Jean Mayer USDA Human Nutrition Research Center on Aging, Tufts University, Boston, MA, USA

**Keywords:** Familial hypercholesterolemia, Coronary artery disease, LDL-receptor mutations, LDL-c goal, combined therapy

## Abstract

**Aim:**

Familial hypercholesterolemia (FH) patients are at high risk for premature coronary heart disease (CHD). Despite the use of statins, most patients do not achieve an optimal LDL-cholesterol goal. The aims of this study are to describe baseline characteristics and to evaluate Lipid Lowering Therapy (LLT) in FH patients recruited in SAFEHEART.

**Methods and Results:**

A cross-sectional analysis of cases recruited in the Spanish FH cohort at inclusion was performed. Demographic, lifestyle, medical and therapeutic data were collected by specific surveys. Blood samples for lipid profile and DNA were obtained. Genetic test for FH was performed through DNA-microarray. Data from 1852 subjects (47.5% males) over 19 years old were analyzed: 1262 (68.1%, mean age 45.6 years) had genetic diagnosis of FH and 590 (31.9%, mean age 41.3 years) were non-FH. Cardiovascular disease was present in 14% of FH and in 3.2% of non-FH subjects (P < 0.001), and was significantly higher in patients carrying a null mutation compared with those carrying a defective mutation (14.87% vs. 10.6%, respectively, P < 0.05). Prevalence of current smokers was 28.4% in FH subjects. Most FH cases were receiving LLT (84%). Although 51.5% were receiving treatment expected to reduce LDL-c levels at least 50%, only 13.6% were on maximum statin dose combined with ezetimibe. Mean LDL-c level in treated FH cases was 186.5 mg/dl (SD: 65.6) and only 3.4% of patients reached and LDL-c under 100 mg/dl. The best predictor for LDL-c goal attainment was the use of combined therapy with statin and ezetimibe.

**Conclusion:**

Although most of this high risk population is receiving LLT, prevalence of cardiovascular disease and LDL-c levels are still high and far from the optimum LDL-c therapeutic goal. However, LDL-c levels could be reduced by using more intensive LLT such as combined therapy with maximum statin dose and ezetimibe.

## Background

Heterozygous familial hypercholesterolemia (FH) is the most common genetic disorder associated with the development of premature CHD with an autosomal dominant mode of inheritance, and a prevalence of one case in 400 to 500 in the general population [1]. The disorder is caused by mutations in the gene that encodes the low-density lipoprotein receptor (LDL-r), leading to an increase in plasma low-density lipoprotein cholesterol (LDL-c) levels. To date, more than 800 different functional mutations of the LDL-r gene have been described worldwide, and more than 200 have been documented in Spain [2]. Life expectancy is shortened by 20 to 30 years in FH patients[3], and sudden death and myocardial infarction are the principal causes of death [4,5]. The genetic defect is probably the most important factor in the clinical expression of FH; however, other genetic, environmental and metabolic factors could play an important role in modulating the atherosclerotic burden in this population[6-8]. Since 1990's, coronary mortality and total mortality in FH patients have markedly decreased in part due to the use of statins [9-11]. However, despite the use of statins, most of these patients do not achieve an optimal therapeutic LDL-c level [12] and still have a high risk for the development of premature CHD.

The SpAnish Familial HypErcHolEsterolaemiA CohoRt STudy (SAFEHEART), was designed to gain insight into the prognostic factors and mechanisms that influence the development of CHD and mortality in a well-defined FH population. The aims of this study are to describe baseline characteristics and to evaluate Lipid Lowering Therapy (LLT) in FH patients recruited in SAFEHEART.

## Methods

### Study Design and Subjects Recruitment

SAFEHEART is an open, multicenter, long-term prospective cohort study in a well-defined FH population, conducted in nineteen outpatient lipid clinics in Spain. Recruitment of subjects from FH families began in 2004 and is still ongoing. Inclusion criteria are: 1) index cases (IC) with genetic diagnosis of FH, 2) relatives over 15 years old with genetic diagnosis of FH, 3) relatives over 15 years old without a genetic diagnosis of FH (control group). Relatives with high cholesterol due to other causes in the control group were not excluded.

To assure and improve the quality of data, physicians attended to 3 different meetings where the different questionnaires and procedures were carefully explained. This study was approved by the local ethics committees and all eligible subjects gave written informed consent.

### Follow-up

A Coordinating Centre was created to organize and to implement the follow-up of cases. A standardized phone call is made every year to the subjects to know any relevant changes in lifestyles, medication, and cardiovascular events. An active epidemiological surveillance system has been developed for the detection of fatal and non fatal cardiovascular events that are classified according to WHO-MONICA criteria [13].

Premature CHD is defined if one of the following events occurs in males before 55 years old and in females before 65 years old, 1) Myocardial infarction, proved by at least two of the following: classic symptoms (> 15 minutes); specific electrocardiographic changes; elevated cardiac enzymes (> 2× upper limit of normal). 2) Angina pectoris, diagnosed as classic symptoms in combination with at least one unequivocal result of one of the following: exercise test, nuclear scintigram, dobutamine stress ultrasound scan, > 70% stenosis on a coronary angiogram. 3) Percutaneous coronary intervention or other invasive procedures and coronary artery bypass grafting.

### Measures and blood samples

Demographic and clinical characteristics of subjects include: age, educational status, medical history focused on cardiovascular disease (CVD), classic cardiovascular risk factors (hypertension, type 2 diabetes, smoking status), physical examination and current treatment for hypercholesterolemia and other risk factors. History of CVD is obtained from medical charts provided by the subjects at inclusion. A quality of life (SF-12) [14], food frequency [Vázquez C, Alonso R, Garriga M, de Cos A, de la Cruz JJ, Fuentes-Jiménez F, Mata P. Validation of a food frequency questionnaire in Spanish patients with Familial Hypercholesterolaemia. Nutr Metab Cardiovasc Dis, in press] and physical activity [15] surveys are recorded on standardized form. Physical examination includes weight (kg), height (cm), body mass index (Kg/m^2^) and waist circumference (cm). Blood pressure is measured twice in supine position with an Omron MX3 sphyngomanometer.

Venous blood samples are taken after 12 hours of fasting. Serum, plasma and DNA samples are aliquotted and preserved at -80°C in a biobank located at the Cardiovascular Research Center in Barcelona. DNA is isolated from whole blood using standard methods and the genetic diagnosis of FH is made using a DNA-microarray^2 ^(Progenika SA, Bilbao, Spain). Serum total cholesterol, triglycerides and HDL-cholesterol levels are measured in a centralized laboratory using enzymatic methods. Serum LDL-c concentration is calculated using the Friedewald formula [16].

Mutations were classified as receptor-negative or receptor defective depending on their functional class as reported previously[7]. Those mutations with non reported functional class in the literature were classified as "unknown".

### Lipid lowering therapy classification (LLT)

Maximum statin dose was defined as previously described [12]: simvastatin 80 mg/day, pravastatin 40 mg/day, lovastatin 80 mg/day, fluvastatin 80 mg/day, atorvastatin 80 mg/day, rosuvastatin 20-40 mg/day. Maximum statin (MS) dose combined with ezetimibe 10 mg/day was considered as maximum combined therapy (MCT). The following treatment was considered giving at least a 50% reduction in LDL-c baseline-levels: simvastatin 20 or 40 or 80 mg/day in combination with ezetimibe 10 mg/day, pravastatin 40 mg/day in combination with ezetimibe 10 mg/day, fluvastatin 80 mg/day in combination with ezetimibe 10 mg/day, atorvastatin 40 or 80 mg/day, atorvastatin 10 or 20 mg/day in combination with ezetimibe 10 mg/day, rosuvastatin 20 or 40 mg/day and rosuvastatin 10 mg/day in combination with ezetimibe 10 mg/day.

### Statistical analysis

An initial descriptive analysis was carried out using number of cases and percentages for qualitative variables and mean and standard deviation for quantitative variables with a normal distribution. For those quantitative variables with a non normal distribution, median and interquartilic-range were estimated. Comparisons of frequencies between qualitative variables were carried out using the Chi-squared test. Mean values of quantitative variables were compared with the Student's t-test for independent data while median values were compared with the non-parametric median test. The relationship between variables was considered statistically significant if the p value < 0.05.

A binary logistic regression analysis (forward) was conducted to determine the variables associated with the attainment of an LDL-c < 100 mg/dl, including those variables proved to be statistically significant in bivariate analyses and possible confounders (age, gender, tobacco, diabetes mellitus, high blood pressure, presence of CAD, type of mutation, type of LLT). The magnitude of the association was estimated using the adjusted odds ratio (OR) with a confidence interval (CI) of 95%. All statistical analyses were performed using the Statistical Package for the Social Sciences (SPSS v 18.0, Chicago, IL, USA).

## Results

**Figure 1:**
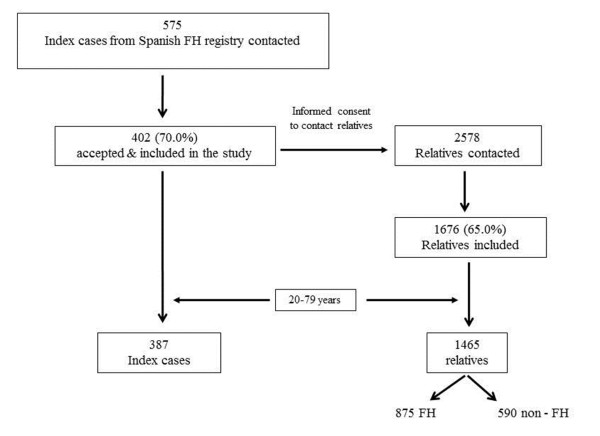
**Recruitment of cases in the SAFEHEART study**.

Up to 2010, the study has enrolled 2078 participants (1402 FH cases and 676 unaffected relatives from 402 families (Figure 1). Baseline clinical characteristics and lipid profile of 1852 participants between 20 to 79 years are shown in Table 1. No data for subjects aged < 20 years are shown because no previous history of CVD was observed. There were no differences in gender between FH and control subjects. Mean age was 45.6 years (SD: 14.7) in FH subjects and 41.3 years old (SD: 14.7) in non FH subjects (p < 0.001).

**Table 1:** Baseline characteristics of subjects recruited in SAFEHEART (20 to 79 years old, N = 1852)

	Index Cases N = 387	FHR (+) N = 875	FHR (-) N = 590	P-value
Gender (male)%	52.2	46.3	46.3	0.117
Mean age (years) * (Age range)	49.1 ± 12.9 (20.2-78.0)	44.1 ± 15.2 (20.1-79.0)	41.3 ± 14.7 (20.2-78.5)	< 0.001
Previous Clinical Diagnosis of FH (%)	100	74.6	13.9	< 0.001
History of CVD (%)	21.7	10.6	3.2	< 0.001
Age of first Cardiovascular event (Age range)	42.8 ± 9.7 (24.0-67.0)	50.3 ± 11.6 (28.0-76.0)	53.9 ± 8.5 (39.0-69.0)	< 0.001
Previous LLT (%)	96.9	77.8	18.8	< 0.001
Type 2 Diabetes (%)	2.1	4.0	3.6	0.220
High Blood Pressure (%)	14.7	14.6	12.7	0.531
Tobacco (current/past)(%)	20.2/33.3	32.0/21.0	34.6/17.1	< 0.001
Xanthomas (%)	20.7	12.8	0.0	< 0.001
BMI (Kg/m^2^) *	26.7 ± 4.3	26.6 ± 5.00	26.2 ± 5.2	0.272
Total cholesterol (mg/dL)*	256.6 ± 71.9	264.6 ± 69.6	213.3 ± 46.4	< 0.001
Triglycerides (mg/dL)**	89.0 (52)	85.0 (56)	85.0 (54)	0.239
LDL-C (mg/dL)*	188.0 ± 69.9	193.8 ± 66.9	131.3 ± 41.4	< 0.001
HDL-C (mg/dL)*	48.6 ± 13.6	50.3 ± 13.9	51.1 ± 13.9	0.001

FH, Familial Hypercholesterolemia; FHR, Familial Hypercholesterolemia relatives; CVD, Cardiovascular Disease; LLT, Lipid lowering therapy; BMI, Body Mass Index; LDL-C, cholesterol transported on low-density lipoproteins; HDL-C, cholesterol transported on High Density Lipoproteins. * values are expressed as Mean and standard deviation; ** values are expressed as Median and interquartilic range.

### Clinical and molecular diagnosis

Most of the IC (88.3%) and FH relatives (73.3%) have been clinically diagnosed by the specialist. However, 25.4% of relatives with molecular diagnosis of FH (mean age 44.8 years, SD 15.9) did not know their clinical condition. Respect to genetic diagnosis, 120 different functional mutations in LDL-r and Apo B genes have been identified in this cohort and 25 mutations represent 65.0% of the cases recruited. Type of mutation was classified as receptor-negative or null mutations in 56.3% of FH subjects, while 35.2% had defective mutations.

### Classical risk factors and cardiovascular disease

Prevalence of current smokers was 28.4% in FH subjects and 34.6% in the control group (P < 0.001). There were no significant differences in the prevalence of type 2 diabetes and hypertension between both groups. HDL-c levels were lower in FH patients compared with controls (P < 0.001) and there were no differences between FH subjects with null and defective LDL-r mutations (49.1 ± 11.8 and 51.2 ± 14.3 mg/dl, respectively, P = 0.208).

History of CVD was present in 14.0% of FH patients, almost 5 times the prevalence found in non-FH subjects (p < 0.001). Coronary heart disease was the most frequent CVD (93.4%) and it was premature in 80.0% of cases. First cardiovascular event occurred earlier in index cases (IC) compared with FH and non-FH relatives (P < 0.001) (Table 1). Prevalence of cardiovascular disease, especially CHD was higher in patients carrying null mutations than those carrying defective mutations in LDL-r gene (14.8% vs. 10.6% respectively, P < 0.05).

### Lipid lowering therapy

At inclusion, 1056 (83.7%) FH cases (97.0% of IC and 78.0% of relatives) were receiving LLT compared to 18.8% of non-FH subjects (p < 0.001). Nearly all cases on LLT were receiving statins (95.4%), either in monotherapy (58.3%) or in combined therapy (37.0%), especially with ezetimibe (31.3%). The most frequent prescribed statins were Atorvastatin (57.3%), Simvastatin (28.9%) and Rosuvastatin (2,9%). Mean doses of statins were: Atorvastatin 44.8 mg (SD: 25.8), Simvastatin 40.7 mg (SD: 23.3) and Rosuvastatin 28.8 mg (SD: 12.7). Only 13.4% of FH subjects were receiving MS dose, and 13.6% were receiving MCT.

### LDL-c levels and LDL-c goal achievement

LDL-c level was not available in 91 FH cases.

Mean LDL-c level was 220.7 mg/dl (SD: 72.5) in those FH cases that were not receiving LLT (16.3%) compared to 186.5 mg/dl (SD: 65.6) of LDL-c level in FH cases on LLT (83.7%). Of those FH patients receiving LLT, only 33 (3.4%) had an LDL-c level < 100 mg/dl, while 149 (15.5%) had an alternative LDL level < 130 mg/dl. On the other hand, 270 FH patients (29.0%) receiving combined therapy (of whom 47.7% were on MCT) did not reach LDL-c goal. Cases with an LDL-c > 160 mg/dl and > 200 mg/dl in the MCT group were 54 (41.5%) and 21 (16.1%), respectively. LDL-c levels in patients using combined therapy with ezetimibe was slightly higher but not significant in those FH subjects with defective mutation compared with null mutation (171.2 ± 67.6 vs. 161.2 ± 57.2, respectively, P = 0.227).

Characteristics of FH patients according to attainment of LDL-c goal are shown in Table 2. Patients with an LDL-c on target were older, had a higher prevalence of CVD history, type 2 diabetes mellitus, hypertension, and were mostly treated with combined therapy than those subjects not on target (p < 0.05). The percentage of cases receiving LLT expected to reduce LDL-c levels at least 50% was 51.5% and was higher (63.6%) in patients on goal compared to those patients not on target (49.9%), although this difference was not statistically significant. However, LDL-c levels were lower in those patients with more intensive LLT (174.0 ± 62.2 mg/dl vs. 196.5 ± 63.8 mg/dl in patients receiving medication to reduce LDL-C ≥50% or below, respectively). There were no differences in LDL-c goal attainment between cases with null and defective mutations. The attainment of LDL-c goal was slightly higher but not statistically significant in those cases treated by specialists compared to general practitioners (59.3% vs 55.2%, P = 0.677).

**Table 2:** Characteristics of FH patients on LLT classified according to attainment of LDL-c goal

	LDL-Ch < 100 mg/dL (N = 33)	LDL-Ch ≥ 100 mg/dL (N = 932)^A^	P-value
Male gender, N(%)	18 (54.5)	454 (48.7)	0.510
Cases ≥ 45 years, N (%)	24 (72.7)	492 (52.8)	0.024
Age (y)*	54.7 (SD:14.5)	47.4 (SD:14.2)	0.004
History of CVD, N (%)	10 (30.3)	153 (16.4)	0.036
Type 2 Diabetes, N (%)	4 (12.1)	33 (3.5)	0.012
High Blood Pressure, N (%)	10 (30.3)	157 (16.8)	0.045
Current smokers, N (%)	5 (15.2)	239 (25.6)	0.173
BMI (Kg/m^2^)*	26.6 (SD:4.0)	26.7 (SD:4.6)	0.882
Control by Specialist, N (%)	16 (59.3)	293 (53.2)	0.677
Null/Defective mutations, N (%)	16/14(48.5/42.4)	541/311(58.0/33.4)	0.525
Years on statin treatment*	7.9 (SD:8.1)	6.8 (SD:6.3)	0.334
≥ 50% LDL-c reduction, N (%)	21 (63.6)	471 (49.9)	0.254
Monotherapy with Statins, N (%)	11 (33.0)	565 (60.6)	0.002
Maximum Statin dose, N (%)	1(3.0)^B^	134 (14.4) ^B^	0.229
Combined Therapy, N (%)^C^	18 (54.5)	270 (29.0)	0.002
Maximum Therapy, N (%)^D^	6 (18) ^B^	124 (13.3) ^B^	0.238

FH, Familial hypercholesterolemia; LLT, Lipid-lowering therapy; CVD, Cardiovascular disease; BMI (Body mass index) (A) LDL-ch levels were uknown in 91 cases; (B) percentage calculated with total cases in each group. (C) Combined therapy includes only any statin + ezetimibe 10 mg; (D), Maximum therapy includes any statin at maximum dose + ezetimibe 10 mg; *Mean and Standard Deviation (SD).

Logistic regression analysis showed that combined therapy using statin and ezetimibe was significantly associated with the achievement of LDL-c goal (OR adjusted: 2.22; IC95%:1.10-4.48), after adjustment for age, sex, CVD and cardiovascular risk factors.

## Discussion

SAFEHEART is a long-term prospective cohort of a molecularly well-defined FH population in Spain. The present study shows that although most of FH cases are on LLT, cardiovascular risk is still high, in part due to the high LDL-c levels. An LDL-c level below 100 mg/dl was reached by 3.4% of patients receiving treatment, and of those patients not at target, 71% were not receiving combined therapy with ezetimibe.

Compared with other FH prospective studies [5,9-11], this cohort establishes genetic diagnosis of all study participants, eliminating misclassification bias when clinical criteria that may include polygenic hypercholesterolemia or familial combined hyperlipidemia are used. It has been demonstrated that more than 30% of FH cases might be classified incorrectly if only clinical diagnosis is used compared with the use of genetic test [17]. In this cohort, the DNA test identified 25,4% of relatives that did not know their clinical condition, with a mean age of 45 years, that is higher than the age recommended for starting LLT in agreement with clinical guidelines [18]. DNA testing offers a definitive and specific diagnosis and those systems based on a DNA microarray have been demonstrated to be cost-effective [19]. This study confirms that Spanish FH subjects are genetically very heterogeneous as previously described [2,7].

History of CVD was present in 14.0% of FH patients, almost 5 times the prevalence found in non-FH subjects. This high cardiovascular risk is explained in part by the genetic background and by the presence of other cardiovascular risk factor such as tobacco consumption. This study shows that FH subjects with null mutation have higher prevalence of PCVD as previously described [7]. Prevalence of current smokers is still very high in these subjects (28.4%) and similar to the Spanish general population (29.5%) [20]; therefore, it is necessary to implement appropriate early-intervention strategies based on therapeutic and lifestyle changes, specially focus on quitting smoking.

Another important factor that can affect the development of CHD is related to LLT. The Simon Broom Registry showed that mortality in FH was reduced significantly after the introduction of statins [9]. Besides, a recent publication showed that FH patients without clinical CVD that started statin therapy after 1990 had a 76% reduction in the risk of cardiovascular events although the dose of statin used was lower than the recommended in this population; however, there are no data for LDL-c goal attainment [11].

International Guidelines consider FH patients at high cardiovascular risk and therefore the optimal LDL-c goal should be < 100 mg/dl [21,22] or to achieve at least a 50% reduction in LDL-c levels [18]. This study shows that although most of FH Spanish patients are treated with LLT, only 3.4% have an LDL-c below 100 mg/dl; however, half of this population were receiving treatment to reduce LDL-C ≥ 50%. The best predictor for goal attainment was the use of combined therapy with ezetimibe. A large cross-sectional study carried out in The Netherlands showed that 21% of FH patients achieved LDL-c goal [12]. This difference could be explained in part because 25% of Dutch patients were on maximum therapy, whereas this occurred only in 13.6% of Spanish patients. On the other hand, 71% of the cases that were not on target were not receiving combined therapy. The use of combined therapy with ezetimibe in this cohort is low, probably because until now there is no evidence of the benefit on atherosclerotic burden measured as intima-media thickness in this population [23]. However, it has been shown that co-administration of ezetmibe with a statin is effective in reducing the estimated risk for cardiovascular mortality measured by the Framingham model in hypercholesterolemic patients [24]. The recent ezetimibe's reimbursement approval for FH patients and the introduction of rosuvastatin in Spain will probably contribute to increase the attainment of LDL-c goal, or at least a more intensive LDL-c reduction.

Although this is a cross-sectional analysis, the long-term follow-up of this cohort will allow to determine the role of changes in lifestyle and the further effect of a more intensive LLT and LDL-c goal attainment in the reduction of cardiovascular morbidity and mortality. The results of this study show that there is still room for improvement in FH treatment in terms of using more combined therapy and higher doses of statins.

## Conclusions

In conclusion, most of this high risk FH population still have high LDL-c levels and did not achieve the optimum LDL-c target as recommended by international guidelines. The combination of a statin and ezetimibe was associated with the best attainment of LDL-c goal. There is a medical need for new LLT options in combination with current therapy to reach lower LDL-c levels together with a better control of other risk factors to prevent CAD in this population.

## List of Abbreviations

FH: Familial hypercholesterolemia; IC: Index case; CHD: Coronary heart disease; LLT: Lipid lowering therapy; LDL-r: Low-density lipoprotein receptor; CVD: cardiovascular disease

## Competing interests

The authors declare that they have no competing interests.

## Authors' contributions

NM, RA, LB, FPJ, JLM, EG, JO, PM, provided support in the design of the study. NM, RA, PM, LB, FPJ, coordinated the execution of the project. NM and RA wrote the manuscript.

RA, FF, OM, JLD, JIV, MAB, MP, JFS, LI, PM, collection and data interpretation. NM, RA, EG, performed statistical analysis and data interpretation. LB, TP are responsible of the biobank, sample managing and DNA obtention. FPJ, JLM, FF are responsible for biochemical analysis.

All authors' read and corrected draft version and approved the final manuscript

## Funding

This study was supported by Fundación Hipercolesterolemia Familiar and grants G03/181, Hiperlipemias Genéticas Network from the Instituto de Salud Carlos III; and grant 08-2008 from Centro Nacional de Investigaciones Cardiovasculares (CNIC).

## Appendix

### The SAFEHEART study group

Fundación Jiménez Díaz, Madrid: PM, RA. Health Promotion and Epidemiology, Spanish Ministry of Health and Fundación Hipercolesterolemia Familiar, Madrid: NM. Centro de Investigación Cardiovascular CSIC-ICCC, Hospital Sant Pau and IIB-Sant Pau, Barcelona: LB, TP. IMIBIC/Hospital Reina Sofía, Córdoba: FF, FPJ, JLM. Hospital Virgen del Rocío, Sevilla: OM, J. Villar-Ortiz. Hospital Abente y Lago, A Coruña: JLD. Hospital de Lugo, Lugo: JIV, R. Argueso. Hospital San Pedro de Alcántara, Cáceres: JFS. Hospital San Pedro, Logroño: A. Brea, D. Mosquera. Hospital de Vitoria, Vitoria: LI. Hospital Comarcal Vega Baja, Orihuela: JM Cepeda. Hospital General de Albacete, Albacete: MAB. Hospital de Elche, Alicante: MP, AM Hidalgo. Hospital Central de Asturias, Oviedo: P Gómez-Enterría, C Martínez-Faedo. Hospital de Mérida, Badajoz: P Saenz. Hospital Clínico, Barcelona. D. Zambón. Hospital Ramón y Cajal, Madrid: C Vázquez, F. Arrieta. Hospital de Donostia, Donostia: F. Almagro. Hospital Ciudad Real, Ciudad Real: J Galiana. Hospital de Igualada: J. Panisello. Hospital Nuestra Señora de la Candelaria, Tenerife: F Pereyra, M Muros.
